# Cloned genes and genetic regulation of anthocyanin biosynthesis in maize, a comparative review

**DOI:** 10.3389/fpls.2024.1310634

**Published:** 2024-01-24

**Authors:** Zaid Chachar, RuiQiang Lai, Nazir Ahmed, Ma Lingling, Sadaruddin Chachar, Najeeba Parre Paker, YongWen Qi

**Affiliations:** ^1^ College of Agriculture and Biology, Zhongkai University of Agriculture and Engineering, Guangzhou, China; ^2^ Guangdong Laboratory for Lingnan Modern Agriculture, Guangzhou, China; ^3^ College of Horticulture and Landscape Architecture, Zhongkai University of Agriculture and Engineering, Guangzhou, China; ^4^ College of Agriculture, Jilin Agricultural University, Changchun, Jilin, China; ^5^ Department of Plant Sciences, Qaid Azam University, Islamabad, Pakistan

**Keywords:** anthocyanins, maize, SH2, ZmCOP1, ZmHY5

## Abstract

Anthocyanins are plant-based pigments that are primarily present in berries, grapes, purple yam, purple corn and black rice. The research on fruit corn with a high anthocyanin content is not sufficiently extensive. Considering its crucial role in nutrition and health it is vital to conduct further studies on how anthocyanin accumulates in fruit corn and to explore its potential for edible and medicinal purposes. Anthocyanin biosynthesis plays an important role in maize stems (corn). Several beneficial compounds, particularly cyanidin-3-O-glucoside, perlagonidin-3-O-glucoside, peonidin 3-O-glucoside, and their malonylated derivatives have been identified. C1, C2, Pl1, Pl2, Sh2, ZmCOP1 and ZmHY5 harbored functional alleles that played a role in the biosynthesis of anthocyanins in maize. The Sh2 gene in maize regulates sugar-to-starch conversion, thereby influencing kernel quality and nutritional content. ZmCOP1 and ZmHY5 are key regulatory genes in maize that control light responses and photomorphogenesis. This review concludes the molecular identification of all the genes encoding structural enzymes of the anthocyanin pathway in maize by describing the cloning and characterization of these genes. Our study presents important new understandings of the molecular processes behind the manufacture of anthocyanins in maize, which will contribute to the development of genetically modified variants of the crop with increased color and possible health advantages.

## 1 Introduction

### 1.1 Background

Anthocyanins are vibrant water-soluble pigments belonging to the flavonoid class, imparting red, purple and blue hues to flowers, fruits, leaves, and stems of plants (Alappat and Alappat, 2020; Lin et al., 2023). Beyond their aesthetic contribution these pigments play crucial roles in plant biology influencing genetic and physiological aspects (Mannino et al., 2021). Essential for a plant’s fitness they aid in antioxidant protection defense against herbivores, pathogens and assist in attracting pollinators. Additionally anthocyanins are pivotal in regulating plant growth and development and in mitigating oxidative damage caused by environmental stressors such as UV radiation and infections (Krga and Milenkovic, 2019; Oliveira et al., 2020; Zhang et al., 2014).

The incorporation of anthocyanins in fruits and vegetables enhances their nutritional value and shelf-life offering significant health benefits when consumed by humans. These benefits include antioxidant properties and potential anti-inflammatory effects (Shipman et al., 2021; Khoo et al., 2017; Speer et al., 2020; Zhang and Zhu, 2023). In agriculture, anthocyanin-rich maize demonstrates increased resilience to environmental stresses and pests potentially reducing the reliance on chemical pesticides and promoting sustainable farming practices (Câmara et al., 2022).

Economically the demand for health-promoting naturally pigmented foods positions anthocyanin-enriched maize as a valuable crop. In the food industry these maize varieties serve as natural colorants aligning with the growing trend for clean-label products (Belwal et al., 2020). Furthermore the cultivation and study of anthocyanin-rich maize present opportunities for scientific exploration in dietary benefits plant health, food science and medicine. Culturally the unique colors and potential flavors of this maize variety offer new culinary experiences enhancing the cultural significance of food (Khoo et al., 2017).

Anthocyanins are primarily found in the glycoside form as anthocyanins and in the aglycone form as anthocyanidins with common variants including peonidin, petunidin, pelargonidin, delphinidin, cyanidin and malvidin (Zhang and Zhu, 2023). They are synthesized via the phenylalanine pathway part of the broader flavonoid pathway as recent studies have shown (Wu Y. et al., 2023). The synthesis of anthocyanins is regulated at the transcriptional level involving transcription factors (TFs) and structural genes. MicroRNAs (miRNAs) also play a significant role in this process by targeting and suppressing TFs thereby affecting the expression of genes crucial for anthocyanin synthesis (Naing and Kim, 2018; Y. Wu et al., 2023). Our review aims to provide a comprehensive overview of plant anthocyanin biosynthesis focusing on the pathway of biosynthesis TFs involved in regulation the impact of environmental factors and the influence of external hormones. This approach seeks to enhance the understanding of anthocyanin research and serve as a foundation for future studies in this field (Y. Zhao et al., 2022).

In maize (Zea mays L.) a key cereal crop globally, anthocyanin biosynthesis reflects a complex genetic interplay resulting in diverse pigmentation and phenotypic traits (Rouf Shah et al., 2016). This review will thoroughly characterize the anthocyanin production pathway in maize emphasizing the functions and regulatory mechanisms of genes such as C1, C2, Pl1, Pl2, Sh2, ZmCOP1 and ZmHY5 (Curtis Hannah et al., 2012; Piazza et al., 2002; Huai et al., 2020). It identifies a research gap in the comprehensive understanding of the genetic network controlling anthocyanin biosynthesis in maize. Despite detailed characterizations of specific genes like Sh2 and ZmCOP1/ZmHY5 a broader understanding of molecular mechanisms and regulatory dynamics is lacking. This gap underscores the need for further investigation into the genetic factors influencing anthocyanin production and plant traits. Moreover the review underlines the potential of developing genetically improved maize varieties for health benefits while also noting the current lack of practical application in breeding and agricultural practices.

### 1.2 The evolution of anthocyanin research

The rising significance of anthocyanins in various sectors along with their potential health benefits has led to a surge in research focused on developing effective affordable and environmentally friendly methods for their extraction. The use of organic solvents in the extraction and purification of anthocyanins has been critically examined due to their harmful environmental impact and negative effects on biological systems. Considerable attention has been devoted to conducting comprehensive investigations of sustainable extraction methodologies. The utilization of supercritical fluids (SCFs) in green extraction procedures has seen significant growth in recent years mostly because of their advantageous environmental attributes (Brglez Mojzer et al., 2016).

Anthocyanins are a class of pigments that have been extensively studied for their biogenetics (Petroni and Tonelli, 2011; Tasaki et al., 2019). Prior to comprehensive studies that validated their numerous bioactivities scholarly investigations primarily centered on the diverse genes involved in the pathway responsible for changing the structure and amplification of pigments. Molecular biological techniques have been used to generate new products that meet the demands of the food beverage and nutraceutical sectors. These products aim to stabilize anthocyanin colors for nutraceutical and food colorant purposes through molecular changes. (Alappat and Alappat, 2020). The growing importance of natural substitutes for food colorants and the heightened recognition of the environmental risks associated with their synthetic counterparts have expedited research efforts in pursuit of this objective. The demand for natural colorants on a global scale has experienced a notable increase over the last decade according to research by (Iorizzo et al., 2020). Consequently numerous food colorant industries have been inspired to make changes to natural food colorants. The main challenge encountered by this industry has been the financial implications of developing a consistent and reliable natural color. This has prompted the pursuit of innovative and financially feasible methods for manufacturing, extraction, refinement and storage of food colorants derived from anthocyanins. The transition from synthetic to natural colorants is contingent on the durability of these pigments in different food matrices and under specified settings. The stability of acylated and co-pigmented anthocyanins has been reported previously (Eiro and Heinonen, 2002; Bakowska-Barczak, 2005). The pigments’ ability to retain their colors across different pH levels and processing circumstances renders them viable candidates for use in dairy products and ready-to-eat desserts. In addition to the above stabilizing approaches, the addition of various chemicals including polymers phenolic compounds, and metals has also been employed. Successful ways to stabilize anthocyanin colors have been explored including the absence of oxygen during processing and storage as well as the encapsulation of pigments. The integration of synthetic and semisynthetic approaches in conjunction with formulation strategies employing novel materials with stabilizing properties has the potential to augment the utility of anthocyanins as natural food colors with added value (Cortez et al., 2017).

### 1.3 Purpose of the review paper

Molecular identification and characterization of the major genes involved in this pathway are the main focus of this review which aims to provide a thorough analysis of the genetic regulation of anthocyanin production in maize. Cloning and Characterization: The primary goal was to present the cloning and detailed characterization of two significant genes Sh2 which plays a pivotal role in regulating sugar-to-starch conversion, and ZmCOP1/ZmHY5 which are key regulatory genes responsible for controlling light responses and photomorphogenesis in maize.

Completion of Genetic Identification: Another important objective is to demonstrate that molecular identification of these genes represents a significant milestone in maize research. By identifying and understanding the role of these genes this review finalizes the identification of all genes responsible for coding the structural enzymes essential in the pathway of anthocyanin biosynthesis in maize.

Insights into Molecular Mechanisms: The intricate molecular mechanisms underlying the biosynthesis of anthocyanins in maize. By elucidating these mechanisms, this study contributes to a deeper understanding of how maize plants produce anthocyanins.

Contribution to Genetic Improvement: The ultimate purpose of this study was to underscore the practical significance of these findings. By shedding light on the genetic regulation of anthocyanin production this review highlights the potential for developing genetically improved maize varieties with enhanced color traits. Additionally it emphasizes the potential health benefits associated with anthocyanin-rich maize varieties. In this review paper’s overarching purpose is to advance our knowledge of maize genetics and anthocyanin biosynthesis offering a foundation for future research and potentially leading to the development of maize cultivars with improved color characteristics and potential health-promoting properties.

## 2 Anthocyanin metabolisms

### 2.1 Molecular regulations of anthocyanin in plant

The regulation of anthocyanin biosynthesis in plants is a complex process that involves multiple molecular components and environmental factors (Do et al., 2023). The transcription factors that regulate the expression of genes encoding the enzymes involved in anthocyanin production are at the focus of this regulation. (Ayvaz et al., 2023). A key group of transcription factors essential in controlling anthocyanin levels is the MYB-bHLH-WD40 (MBW) complex (Yan et al., 2021). This complex is composed of three essential elements MYB transcription factors basic helix-loop-helix (bHLH) transcription factors and WD40 repeat proteins. MYB transcription factors like PAP1, PAP2, and MYB113 are crucial in initiating the activation of genes involved in anthocyanin biosynthesis (He et al., 2023). These factors attach to distinct cis-regulatory elements termed MYB-binding sites, located in the promoters of related genes. bHLH transcription factors like TT8, TTG1 and GL3 collaborate with MYB factors to amplify their function and attract WD40 proteins thereby assembling the functional MBW complex (Pireyre and Burow, 2015; Yang et al., 2022). This complex regulates the expression of genes encoding enzymes such as chalcone synthase (CHS), chalcone isomerase (CHI) and dihydroflavonol 4-reductase (DFR), which are essential for anthocyanin production (Sicilia et al., 2020).

A complex regulatory network varies depending on the specific plant part, environmental conditions, and species. In plant roots anthocyanin production typically responds to stress conditions such as nutrient deficiency and heavy metal exposure which serve as protective responses. This regulation involves a network of transcription factors, including HY5 and MYB75 in Arabidopsis thaliana, which activate key genes encoding enzymes such as chalcone synthase (CHS), chalcone isomerase (CHI) and dihydroflavonol reductase (DFR) which are involved in the anthocyanin biosynthetic pathway (Pesch and Hülskamp, 2004; Dabravolski and Isayenkov, 2023). Recent research has shed light on the intricate regulatory mechanisms governing anthocyanin production in leaves in response to environmental cues such as light exposure and temperature fluctuations. Notably cold temperatures have been identified as triggers of anthocyanin synthesis in certain plant species. At the heart of this regulatory network is the transcription factor HY5 which plays a central role. HY5 activates light-responsive elements located within the promoter regions of genes involved in anthocyanin biosynthesis. Recent investigations have revealed that HY5’s regulatory activity involves its dynamic movement from the shoot to the root. When shoots are exposed to illumination HY5 accumulates and subsequently translocates to the roots in Arabidopsis (Chen et al., 2016). In the root system HY5 exerts control overgrowth and nutrient uptake processes while also promoting its own local expression. Furthermore regulation of anthocyanin production is not solely orchestrated by HY5. Photoreceptors such as phytochromes and cryptochromes also significantly contribute to this intricate regulatory network (Chen et al., 2016; van Gelderen et al., 2018).

Anthocyanins the vibrant pigments found in various parts of plants play a significant role in seed stalks and stems also utilize anthocyanins for protection against UV radiation and herbivores with regulation resembling that in leaves. Light and the HY5 transcription factor are pivotal in this context, whereas MYB transcription factors such as MYB10 and MYB75 serve as key regulators (Yan et al., 2021). At the genetic level the HY5 transcription factor plays a pivotal role HY5 is integral in mediating the plant’s response to light acting as a crucial link between light perception and the biosynthesis of anthocyanins. When plants are exposed to light HY5 is activated and in turn upregulates the expression of genes that are involved in anthocyanin production. Furthermore, the transcription protein levels and function of HY5 are closely regulated by various factors via different control mechanisms (Xiao et al., 2022). They directly activate the genes responsible for the production of these pigments thereby ensuring an efficient and timely response to environmental cues (Shin et al., 2013). The intricate regulation involving light HY5 and MYB transcription factors ensures that plants synthesize anthocyanins effectively enabling them to adapt to varying environmental conditions. This finely-tuned system allows plants to optimally invest resources in producing anthocyanins balancing the energy cost with the crucial need for protection against UV radiation and herbivory. This adaptive response underscores the exceptional capacity of plants to react to their surroundings and safeguard themselves against various external stressors (Ambawat et al., 2013).

Anthocyanin production in plants an integral part of their survival and adaptation mechanisms, extends beyond roots, leaves and stems to encompass a variety of other parts including fruits, flowers and petioles. This complex biosynthetic process is closely intertwined with the plant’s developmental stages environmental stimuli and specific conditions of different tissues (Liu et al., 2018). In the realm of development anthocyanin synthesis is a highly regulated process often associated with the maturation and aging of plant parts. For instance in fruits the onset of anthocyanin production is typically linked with ripening. This is evident in fruits like blueberries and grapes where the accumulation of anthocyanins imparts vibrant colors and also plays a role in attracting seed dispersers (Jaakola et al., 2002). The environmental factors influencing anthocyanin synthesis are diverse. Light is a crucial factor exposure to sunlight can significantly increase anthocyanin levels in plant tissues as seen in the red pigmentation of apples exposed to direct sunlight (Do et al., 2023). Temperature also plays a role with cold temperatures often enhancing anthocyanin production in certain plants such as red lettuce. Additionally water stress and nutrient availability can alter anthocyanin levels often leading to increased synthesis under suboptimal conditions as a protective response against stressors (Li and Ahammed, 2023). Different plant parts synthesize anthocyanins at varying rates and volumes influenced by the unique chemical and hormonal environments of each tissue. For example the anthocyanin composition in flower petals differs from that in leaves contributing to the wide variety of colors in different flowers. This specificity is also a result of the unique gene expression patterns in different tissues which dictate the types and quantities of anthocyanins produced (Mekapogu et al., 2020). Transcription factors from the MYB, bHLH and WD40 protein families often form a complex (MBW complex) to regulate anthocyanin biosynthesis in these tissues (Ma et al., 2021; Yan et al., 2021).

In this study anthocyanin production in plants is a multifaceted process influenced by developmental cues environmental conditions and the specific characteristics of each tissue. Understanding these processes is key to comprehending plant adaptation and survival strategies as well as their aesthetic and nutritional qualities. It is important to acknowledge that the precise genes transcription factors and regulatory elements involved in anthocyanin regulation vary widely among plant species. Beyond transcriptional regulation anthocyanin biosynthesis is also influenced by epigenetic and post-transcriptional mechanisms. Histone acetylation a key epigenetic modification plays a significant role in regulating the expression of genes involved in anthocyanin production. Additionally microRNAs (miRNAs) contribute to the post-transcriptional regulation of these genes. These miRNAs can modulate gene expression by targeting mRNA molecules thereby influencing the anthocyanin biosynthesis pathway and hormone signaling, and other factors add further complexity to the regulation of anthocyanin production. Ongoing research continues to unveil additional details regarding the intricate molecular mechanisms governing this colorful aspect of plant biology (Outchkourov et al., 2018; Cappellini et al., 2021). MYB-bHLH-WD40 (MBW) transcription factor complex a critical regulator in activating anthocyanin biosynthesis across various crop species. Recent advancements have identified additional regulators that either disrupt or stabilize the MBW complex functioning as repressors and activators respectively. These findings add new dimensions to our understanding of the intricate control mechanisms governing anthocyanin production. Moreover it has been discovered that the activity of these repressors and activators is subject to post-translational regulation introducing another layer of complexity in the anthocyanin biosynthetic pathway. This post-translational regulation ensures a more dynamic and responsive control over anthocyanin synthesis adapting to various environmental and developmental signals.

In summary the regulation of anthocyanin biosynthesis in plants is a multifaceted process involving a network of transcription factors repressors and activators within the MBW complex along with layers of post-translational, epigenetic and post-transcriptional control. This complex regulatory network offers multiple potential targets for enhancing anthocyanin content in crops providing exciting opportunities for agricultural and nutritional advancements.

### 2.2 Biosynthesis pathway of anthocyanin

Mostly through the interaction of regulatory genes and plant hormones with the identification of potential key regulatory genes the biosynthesis pathway of anthocyanins has been described in Arabidopsis, tomato, rice and many other species. The biosynthesis pathway of anthocyanins in purple maize has also been well established. Several families of transcription factors and the complex’s regulatory genes primarily control the genes involved in anthocyanin production(Cai et al., 2023). Thus transcription factor activity and structural gene control are essential for the expression of anthocyanins. PAL, C4H, 4CL, CHS, CHI, DFR, LDOX, ANS and UFGT were among the structural genes (Wang et al., 2022). The transcription-related variables MYBs, MYCs, bZIPs, B-box, MYBbHLH-WD40 (MBW) complex, NACs, and WRKYs are important regulators of anthocyanin biosynthesis induction (Sun et al., 2022). It is known that the MBW complex regulates the formation of anthocyanins. Important regulators classified as MYBs have been found in a variety of fruit crops, including grapes, apples, pears, and peaches.(Yan et al., 2021). The accumulation of anthocyanins which are pigments responsible for the red coloration was notably greater in the red-blushed apples compared to their parent varieties. The expression levels of genes involved in anthocyanin synthesis, including MdMYB10, MdMYB3, MdbHLH3, MdWD40 and MdCOL11 (BBX22), varied in relation to the skin color. These variations were particularly evident in patterns of red pigmentation during the 4, 6 and 8-day intervals following the removal of protective bags from the fruit (Vimolmangkang et al., 2013). Glutathione S-transferases (GST) and MATE-type transporters are essential in controlling the movement of anthocyanin from the cytoplasm into the vacuoles. This process significantly enhances the storage of anthocyanin within the vacuolar compartments (D. Jia et al., 2020; Khusnutdinov et al., 2021). Chemically, anthocyanins are polyhydroxy/polymethoxy glycosides derived from anthocyanidins (Cai et al., 2023).

**Figure 1:**
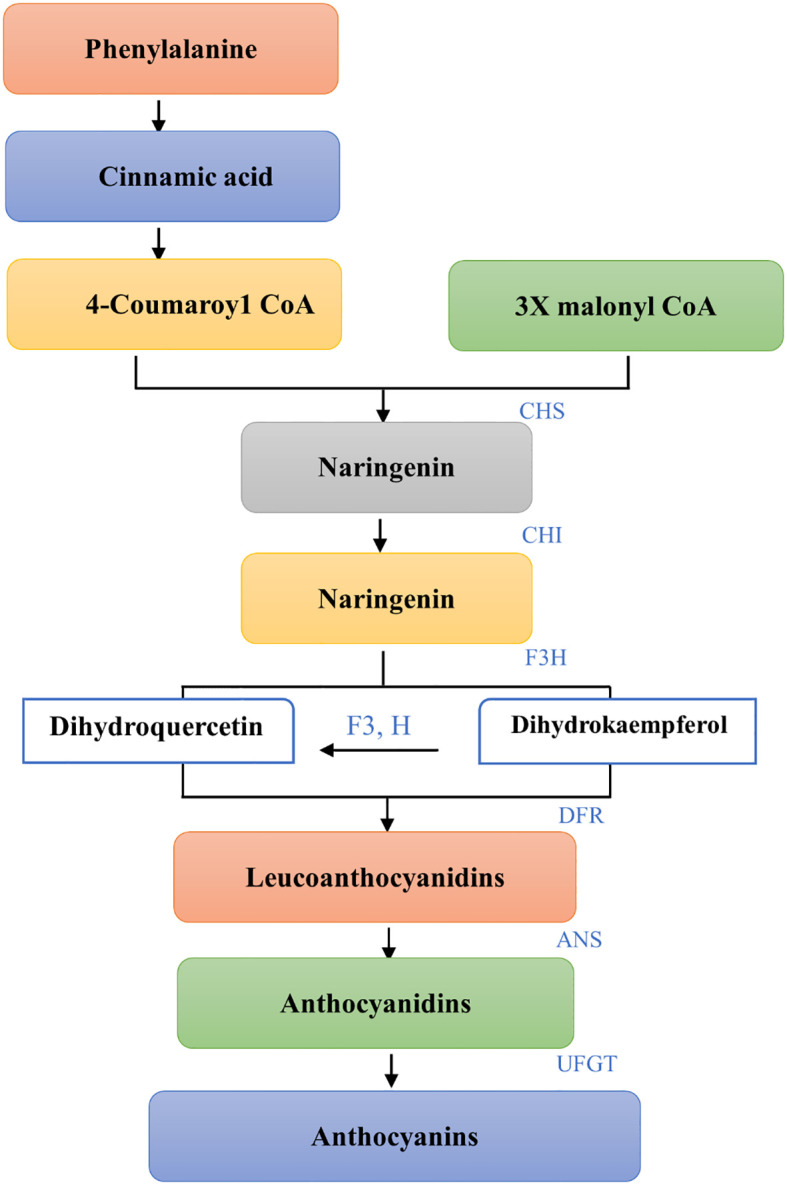
Biosynthetic pathway of Anthocyanins from Phenylalanine. Each step shows the conversion of substrates to products with the specific enzymes involved. The pathway initiates with Phenylalanine leading to Cinnamic acid and proceeds with the formation of 4-Coumaroyl CoA. Coupling with Malonyl CoA under the action of CHS results in Naringenin, which is then hydroxylated by F3H to Dihydrokaempferol and by F3’H to Dihydroquercetin (not shown). Both are reduced by DFR to Leucoanthocyanidins and then converted to Anthocyanidins by ANS. The final step, glycosylation by UFGT, yields Anthocyanins. This schematic representation outlines the enzymatic steps leading to the diverse array of flavonoid pigments that contribute to the coloration in plants.

For purple corn to produce anthocyanins phenylalanine is the first constituent. Prior to being converted into 4-coumaroyl CoA the main precursor of anthocyanins phenylalanine ammonia lyase (PAL) first deaminates phenylalanine to cinnamic acid (Sonmez et al., 2021; Chen X. et al., 2022). Chalcone synthase (CHS) catalyzes the condensation of three malonyl CoA and one 4-coumaroyl CoA molecule to form naringin chalcone, an early critical reaction in the biosynthesis of flavonoids that is typically thought to be the pathway step’s rate-limiting step (Dixon et al., 2002; Yadav et al., 2020). The enzyme chalcone isomerase (CHI) isomerizes naringenin chalcone to naringenin that is colorless. Naringenin is hydroxylated at the third position to produce dihydrokaempferol, which is catalyzed by flavanone 3-hydroxylase (F3H) (DHK). Subsequently, dihydroflavonols’ 3’position is modified by a flavonoid 3’-hydroxylase (F3’H) which can produce dihydroquercetin by using either naringenin or DHK as substrates (DHQ). Colorless Leucoanthocyanidins are produced when Dihydroflavonol-4- reductase breaks down Dihydroflavanols, DHQ and DHK (DFR). Leucoanthocyanidins are further used as substrates by anthocyanidin synthase (ANS) in order to produce anthocyanidins. When flavonoid-3-Oglucosyltransferase (UFGT) catalyzes the colored anthocyanindins for glycosylation the result is the formation of more stable molecules called anthocyanins (as shown in **Figure 1**). The produced anthocyanins will be carried by transporters into the vacuoles where they will be stored as colorful aggregates known as anthocyanin vacuolar inclusions (He et al., 2010; Lago et al., 2013).

### 2.3 Recent advance in anthocyanin integration in maize

More recently anthocyanins have been developed from maize which provides an attractive visual component in addition to increased nutritional value. A group of genes called regulatory genes regulates the biosynthesis of anthocyanins in maize. Key regulatory genes R1 (B-Peru), C1 (Colorless1) and P1 are involved in the anthocyanin production pathway in maize (Purple1) (Sharma et al., 2011). Furthermore, different plant species may have different particular genes involved in the production of anthocyanins. The genetic regulation of anthocyanin synthesis in maize has been thoroughly studied and several important genes involved in this pathway have been cloned and characterized. Despite the fact that the anthocyanin production pathway in different plant species shares some common genes this pathway is largely unique in maize. **Table 1** r epresents the differences and extra genes unique to particular plant families or species. The expression of the structural genes involved in production of anthocyanin pigment is regulated by these transcription factors (Khusnutdinov et al., 2021).

**Table 1:** List of genes involved in the anthocyanin production pathway across different plant species.

Gene	Function	Plant	Description	References
Colorless1 (C1)	Transcription factor	Maize	Regulates the expression of genes involved in anthocyanin synthesis	(Piazza et al., 2002)
Colorless2 (C2)	Transcription factor	–	Forms a complex with C1 to activate anthocyanin biosynthesis genes	(Chen et al., 2022)
Red1 (R1)	Enzyme (DFR)	–	Catalyzes the conversion of dihydroflavonols to anthocyanidins	(Sharma et al., 2012)
Red2 (R2)	Enzyme (ANS)	–	Catalyzes the conversion of anthocyanidin precursors to colored anthocyanins	(Yan et al., 2021)
Purple plant1 (Pl1)	Transcription factor	–	Controls the expression of genes in the anthocyanin pathway	(Sharma et al., 2011)
Purple plant2 (Pl2)	Transcription factor	Maize, Rice	Forms a complex with Pl1 to activate anthocyanin biosynthetic genes	(Gao et al., 2019)
Shrunken2 (Sh2)	Enzyme (ADP-glucose pyrophosphorylase)	–	Encodes an enzyme involved in starch biosynthesis	(Hossain et al., 2015; Chhabra et al., 2019)
MdHY5	Transcription Factor	Maize, Arabidopsis and Apple	Regulating various aspects of plant growth and development, particularly in response to light signals.	(Huai et al., 2020)
ZmCOP1	Regulator of Photomorphogenesis	Maize and Arabidopsis	ZmCOP1 (Zea mays constitutive photomorphogenesis 1) is a gene involved in the regulation of photomorphogenesis in maize	(Huai et al., 2020)
ZmHY5	Transcription Factor	Maize and Arabidopsis	ZmHY5 (Zea mays elongated hypocotyl 5) is a transcription factor gene in maize that plays a crucial role in the regulation of gene expression in response to light.	(Huai et al., 2020)
(MYB) MdMYB3, MdMYB10	Transcription Factor	Apple	Involved in regulation of anthocyanin biosynthesis and flower development	(Vimolmangkang et al., 2013; Liu et al., 2022)
FvDFR2 and FvDFR1	gene	strawberry	play key roles for anthocyanin synthesis in strawberry petioles	(Li et al., 2023)
EsMYB90	Transcription Factor	Eutrema salsugineum, tobacco and Arabidopsis	Enhanced pigmentation and anthocyanin accumulation in various organs	(Qi et al., 2020)
BnaPAP2.A7	Transcription Factor	Brassica napus, canola/rapeseed	Likely involved in the regulation of anthocyanin biosynthesis	(Chen et al., 2020)
AmRosea1and AmDelila	gene	Antirrhinum majus L	Regulation of pigment biosynthesis, for the red, purple, and blue colors in plants.	(Piao et al., 2021)
AtMYB4	Transcription factor regulating phenylpropanoid metabolism	Arabidopsis thaliana	Involved in repressing the phenylpropanoid pathway, affecting lignin biosynthesis and UV protection	(Jin, 2000)
FaMYB1	Regulates anthocyanin and flavonoid biosynthesis	Fragaria × ananassa (Strawberry)	Influences color and flavor by controlling phenolic compound biosynthesis	(Aharoni et al., 2001)
PhMYB27	Likely involved in floral development and pigmentation	Petunia hybrida	May play a role in determining flower color and pattern	(Albert et al., 2014)
VvMYB2-L1	Regulation of proanthocyanidin biosynthesis in grapevine	Vitis vinifera (Grapevine)	Influences the production of proanthocyanidins, impacting grape and wine quality	(Cavallini et al., 2015)
PtMYB182	Potentially involved in secondary metabolism	Populus trichocarpa (Black Cottonwood)	Could be involved in the regulation of lignin and flavonoid biosynthesis	(Yoshida et al., 2015)
MdMYB16	Likely plays a role in apple skin coloration	Malus domestica (Apple)	Influences the pigmentation of apple skin, possibly affecting anthocyanin biosynthesis	(Xu et al., 2017)
AtMYBL2	Transcriptional repressor in anthocyanin biosynthesis	Arabidopsis thaliana	Negatively regulates anthocyanin biosynthesis, impacting coloration	(Dubos et al., 2008)
IlMYBL1	Possibly involved in pigment biosynthesis	Iris lactea var. chinensis	May influence flower coloration through regulation of pigmentation genes	(Matsui et al., 2008)
LhR3MYB1/2	Regulatory genes in flower coloration	Lilium hybrid	Involved in the biosynthesis and regulation of flower pigments	(Gates et al., 2018; Sakai et al., 2019)
AtCPC	Involved in root hair development and trichome formation	Arabidopsis thaliana	Plays a crucial role in epidermal patterning and hair formation	(Zhu et al., 2009)
PhMYBx	May play a role in flower coloration	Petunia hybrida	Potentially involved in the regulation of floral pigmentation and pattern	(Yuan et al., 2013)
SlMYBATV	Involved in anthocyanin biosynthesis	Solanum lycopersicum (Tomato)	Plays a role in regulating anthocyanin accumulation in tomatoes	(Colanero et al., 2018)
FhMYBx	Likely involved in flower pigmentation	Freesia hybrid	Influences the color and pattern of Freesia flowers	(Ding et al., 2020)
MaMYBx	Possibly plays a role in banana fruit ripening and coloration	Musa acuminata (Banana)	May regulate processes related to fruit ripening and pigmentation in bananas	(Li et al., 2020b)
AtSPL9	Regulates plant developmental processes	Arabidopsis thaliana	Involved in the control of plant growth and development	(Zhang et al., 2020)
miR828	MicroRNA involved in the regulation of MYB transcription factors	General in various plants	Plays a regulatory role in plant development and stress responses	(Luo et al., 2012)
miR858	MicroRNA targeting MYB transcription factors	General in various plants	Influences various aspects of plant development and metabolism	(Jia et al., 2015)
AtGL2	Involved in epidermal cell fate determination	Arabidopsis thaliana	Crucial for the development of epidermal structures such as trichomes and root hairs	(Wang et al., 2015)
MdHB1	Likely involved in apple fruit development and ripening	Malus domestica (Apple)	Influences processes related to the growth and maturation of apple fruits	(Jiang et al., 2017)
AtHAT1	Plays a role in plant development and response to light	Arabidopsis thaliana	Involved in light signaling and developmental processes	(Zheng et al., 2019)
AtCOP1-AtHY5	Regulators of photomorphogenesis	Arabidopsis thaliana	Key players in light-mediated developmental processes	(Maier et al., 2013)
SmCOP1-SmHY5	Involved in light signaling and development	Selaginella moellendorffii	Play a role in the regulation of light-dependent developmental processes	(Shin et al., 2013; Jiang et al., 2016)
MdCOP1	Involved in light signaling and development	Malus domestica (Apple)	Plays a role in light-regulated developmental processes in apple	(An et al., 2017)
PpCOP1-PpHY5	Regulate photomorphogenesis	Physcomitrella patens	Involved in the regulation of light-dependent growth and development	(Zhao Y. et al., 2021)
AtLBD37/38/39	Involved in nitrogen metabolism and anthocyanin biosynthesis	Arabidopsis thaliana	Play roles in nutrient response and secondary metabolism	(Rubin et al., 2009)
VvLBD39	Likely involved in grapevine development	Vitis vinifera (Grapevine)	Potentially influences aspects of grapevine growth and fruit development	(Soubeyrand et al., 2014)
MdLBD13	Potentially related to apple development	Malus domestica (Apple)	May play a role in specific developmental processes in apple	(Li et al., 2017)
MdLOB52	Role unclear possibly related to apple development	Malus domestica (Apple)	Could be involved in regulating aspects of apple tree growth and development	(Wang et al., 2018)
AtJUB1	Regulates longevity and stress tolerance	Arabidopsis thaliana	Involved in aging and response to environmental stressors	(Wu et al., 2012)
BoNAC019	Involved in stress response and leaf senescence	Brassica oleracea	Plays a role in the aging of leaves and response to stress conditions	(Wang et al., 2018)
AtTCP15	Controls plant growth and development	Arabidopsis thaliana	Influences various developmental stages and growth patterns	(Viola et al., 2016)
AtJAZ1	Part of the jasmonate signaling pathway	Arabidopsis thaliana	Involved in response to jasmonate, a plant hormone related to stress and defense responses	(Shan et al., 2009)
MdJAZ2	Involved in apple stress response	Malus domestica (Apple)	Plays a role in the apple’s response to environmental stress	(Qi et al., 2011)
MdARF13	Auxin response factor, possibly involved in apple fruit development	Malus domestica (Apple)	Influences how apple trees respond to auxin, affecting fruit growth and development	(Wang et al., 2018)
AtSMXL6	Part of the strigolactone signaling pathway	Arabidopsis thaliana	Involved in the regulation of strigolactones, plant hormones that affect shoot branching	(Liu et al., 2019)
DELLA	Growth repressors, integrate various hormonal signals	General in various plants	Regulate plant growth by integrating hormonal signals, particularly gibberellins	(Jiang et al., 2007)
FaCRY1-FaCOP1-FaHY5	Cryptochrome 1, involved in the blue light photoreception.	Fragaria × ananassa (Strawberry)	influencing seedling growth, flowering, circadian rhythms, photomorphogenesis, and UV-B response,	(Liu et al., 2023)

In maize biology a suite of genes plays a pivotal role in orchestrating key biological processes, particularly in the synthesis of anthocyanins and the regulation of plant growth and development. These genes encompass both structural and regulatory components contributing significantly to the diverse phenotypic traits observed in maize ranging from kernel size and composition to the vivid coloration of various plant parts. Among these, transcription factors such as C1, C2, Pl1, Pl2, Sh2,ZmCOP1, and ZmHY5 are instrumental in regulating the expression of anthocyanin biosynthetic genes (Curtis Hannah et al., 2012; Piazza et al., 2002; Huai et al., 2020). Furthermore genes like A1, A2, R1, Pr, Sh1, Bt1, Bt2, and Su1 highlight the intricate interaction between starch synthesis and anthocyanin production pathways underlining their integral roles in maize’s biological complexity (Cossegal et al., 2008; Mehta et al., 2017).

**Figure 2:**
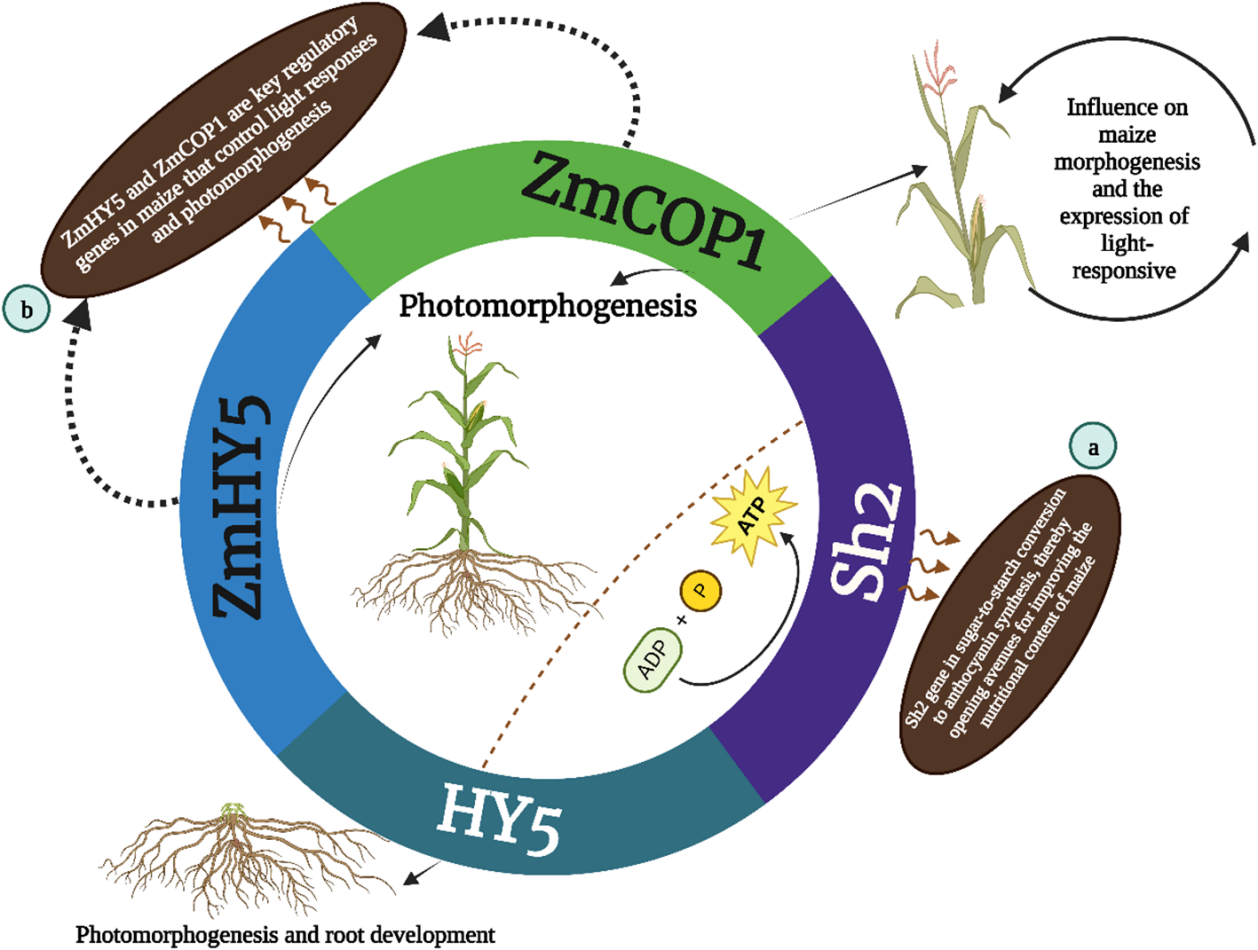
The HY5 gene is involved in photomorphogenesis and root development while the outer annotations (a and b) indicate further specifics: **(A)** Sh2 gene is involved in starch synthesis and has an indirect influence on maize morphogenesis and the expression of light-responsive genes; **(B)** ZmHY5, and ZmCOP1 are key regulatory genes in maize that control light responses and photomorphogenesis.

In parallel ZmCOP1 a gene widely studied across various plant species including Arabidopsis thaliana, Sorghum bicolor and Oryza sativa plays a key role in maize morphogenesis (Huai et al., 2020). Recent research by Chen et al. (2023) has shed light on the functions of ZmCOP1 in maize particularly in its regulation of light-responsive gene expression. This study demonstrates that ZmCOP1 significantly influences the elongation of maize mesocotyl in low-light conditions and affects plant height in light, mirroring the regulatory patterns observed in its Arabidopsis counterpart AtCOP1. The identification of differentially expressed genes (DEGs) in this context underscores ZmCOP1’s role in modulating genes associated with the plant phytohormone pathway, suggesting avenues for enhancing maize performance and growth. Complementing ZmCOP1 and ZmHY5 emerges as another key regulator in maize controlling light responses and photomorphogenesis. Known for its presence in Arabidopsis and various other plant species HY5 governs a range of developmental processes responding to hormonal and environmental cues. Its role in maize particularly in concert with COP1 homologs, accentuates the importance of these genes in plant development and adaptation. The research on ZmCOP1 and ZmHY5 not only elucidates their regulatory influence on maize morphogenesis but also offers valuable insights into potential agricultural applications, particularly in improving crop yields. These findings showed in (**Figure 2B**), that the HY5, ZmHY5 and ZmCOP1are key regulatory genes in maize that control light responses and photomorphogenesis. These discoveries underscore the importance of these genes in maize development and plant biology.

**Figure 3:**
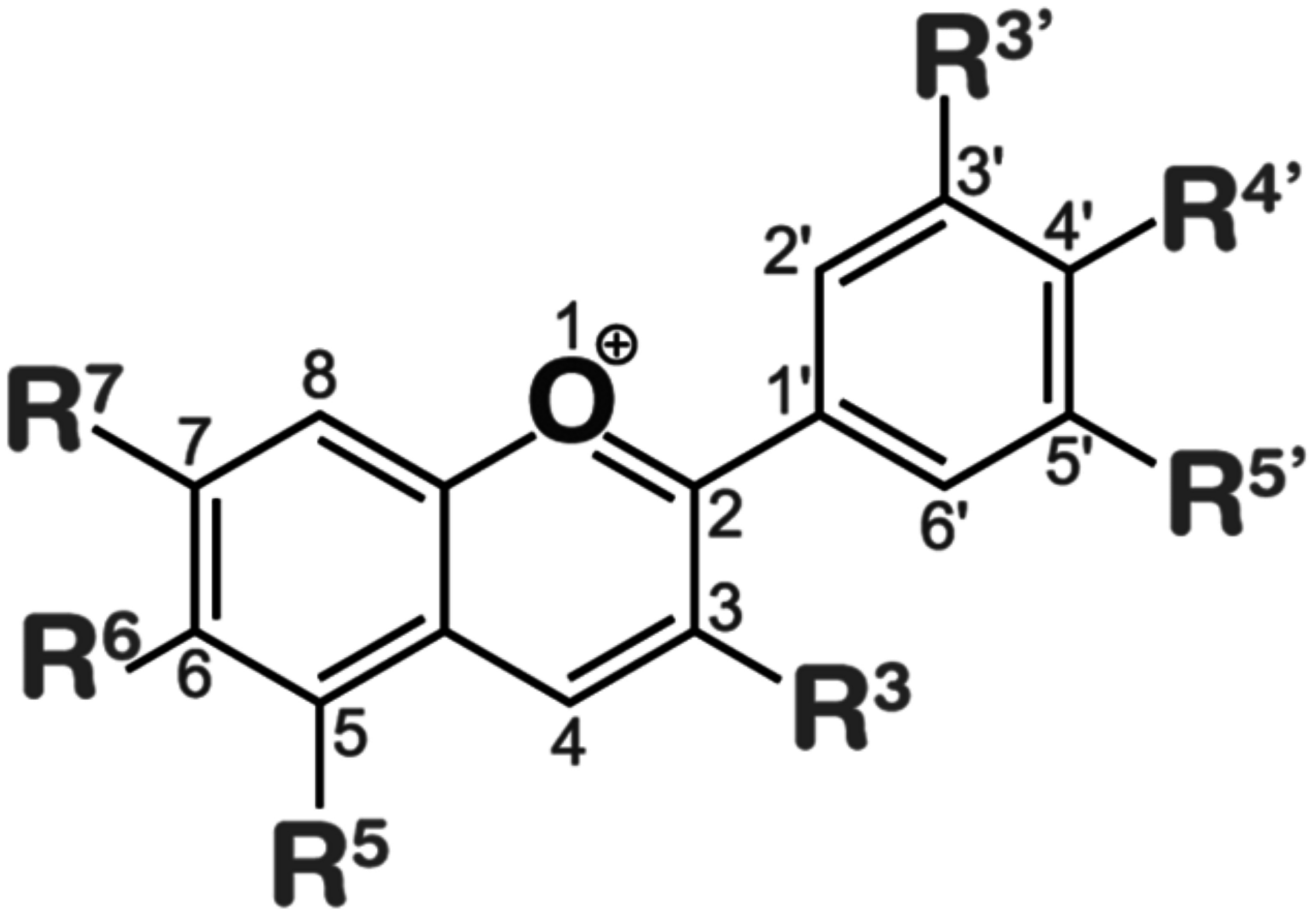
Basic structure of Anthocyanins.

The HY5 protein, found in various plant species, such as Arabidopsis, soybean, pea, apple, moss, tomato, rice and maize, plays a crucial role in regulating the growth of hypocotyls or stems, as well as in responding to both internal signals (such as gibberellic acid and auxin) and external signals (such as light, low temperatures and high temperatures) **Figure 2** showed that the HY5 was initially characterized as a facilitator of photomorphogenesis root gravitropic response and lateral root development in the model organism Arabidopsis as reported by Xiao et al. (2022). According to a recent study by Xiao et al. (2022), the HY5 orthologs of sweet wormwood, sweet orange, strawberry, pear, peach, tomato, eggplant and grape have been found to play a significant role in the regulation of light-induced flavonoid production and accumulation **Figure 3**. HY5 orthologs play a key role in light perception and signal transduction helping plants adjust their growth and development in response to light (Huai et al., 2020; Xiao et al., 2022). HY5 encodes a transcription factor of the bZIP type which exerts regulatory control over approximately one-third of the gene expression across the entire genome (Xiao et al., 2022). Several comprehensive investigations have demonstrated that the regulatory role of HY5 encompasses a wide range of developmental processes and modulation of hormones and environmental signals in plants. These functions are mediated by distinct yet interconnected signaling networks as documented by (Gangappa and Botto, 2016; Su et al., 2021). The protein structures and functions of HY5 orthologs in different plant species exhibit a high degree of conservation as depicted in **Figure 2**. Most plant species have an HY5 protein variant that consists of a basic region and a Leucine Zipper Domain which are important for DNA binding and dimerization respectively. However certain plant species including soybean and pea feature an extra RING-finger motif in their HY5 protein variant according to (Gangappa and Botto, 2016; Xiao et al., 2022). The results of this study show that HY5 orthologs are likely to have both similar and separate roles in the regulation of physiological and developmental processes across different plant species. Orthologs of HY5 in different plant species have been demonstrated to promote various light-regulated developmental processes and responses.

The Sh2 gene identified over four decades ago holds a special place in maize genetics primarily due to its regulatory role in sugar metabolism. It is instrumental in starch biosynthesis catalyzing the conversion of glucose-1-phosphate and ATP to ADP-glucose a substrate critical for starch synthesis and kernel development. The mutation in the Sh2 locus precipitates notable changes in the activity of ADP-glucose pyrophosphorylase a key enzyme in the starch synthesis pathway thereby altering starch production in maize kernels (Bhave et al., 1990). This discovery has had profound implications for understanding the genetic control of starch content in maize endosperm. Anirban et al., 2023 presents the first scientific documentation of breaking the strong genetic linkage between the a1 and sh2 genes in a purple-pericarp super-sweet corn variety. The research began with the creation of five heterozygous purple-pericarp super-sweet corn ears (A1a1.sh2sh2), achieved through breaking this tight genetic linkage via extensive field experiments. Further field trials resulted in the production of the homozygous (A1A1.sh2sh2) purple-pericarp super-sweet corn line named ‘Tim1’. The separation between the a1 and sh2 genes was measured at 0.08 centimorgans (cM) aligning with previous findings in purple-aleurone maize. This recombination break was located in the intergenic space between the a1 and yz1 genes within the a1–yz1-x1–sh2 multigenic interval on chromosome 3. The anthocyanin levels in the newly developed purple-pericarp super-sweet corn line mirrored those of its purple-pericarp maize parent while its sugar profile resembled that of its white super-sweet corn parent. This breakthrough is significant for future breeding programs aimed at producing high-anthocyanin sweet corn offering a nutritionally enhanced food option for consumers (Anirban et al., 2023). Finally (**Figure 2A**) described that the Sh2 gene in sugar-to-starch conversion to anthocyanin synthesis thereby opening avenues for improving the nutritional content of maize. in maize Sh2 regulates sugar metabolism which is crucial for starch biosynthesis and its influence extends beyond mere biochemical pathways impacting kernel development crop yield and the overall quality of the maize thereby holding a significant place in both agricultural practices and crop improvement strategies. Moreover variations in the Sh2 gene can lead to significant differences in kernel composition and characteristics. For instance mutations in Sh2 often result in an increased accumulation of sugars and a corresponding decrease in starch content which is a desirable trait in sweet corn varieties. This altered balance between sugar and starch not only impacts the taste and texture of the corn but also has implications for the crop’s post-harvest qualities, such as shelf-life and processing attributes.

#### 2.3.1 Regulatory mechanisms and functional roles of the MBW complex in anthocyanin biosynthesis and developmental processes in maize (*Zea mays*)

The MBW complex which consists of MYB, bHLH and WD40 proteins plays an important role in the regulation of various biological processes in maize (Zea mays) a critical cereal crop. This transcriptional complex is composed of MYB, bHLH and WD40 proteins which interact to control the expression of genes involved in various aspects of maize growth and development including anthocyanin biosynthesis, trichome formation and kernel development (Cui et al., 2021). Several studies have elucidated the regulatory mechanisms governing the formation and activity of the MBW complex in maize. For example the R/B gene family encodes R2R3-MYB transcription factors that interact with bHLH proteins such as B or Lc to form the MBW complex, which subsequently activates the expression of genes responsible for anthocyanin pigmentation in maize tissues (Lloyd et al., 2017). Additionally the interaction between ZmR, a maize R3-MYB protein, and ZmB, a bHLH protein regulates trichome development in maize leaves (Zimmermann et al., 2004; Huang et al., 2013). These regulatory interactions are critical for maize adaptation and responses to changing environmental conditions as well as for agronomic traits. Further research in this area continues to uncover additional components and nuances of MBW complex regulation in maize shedding light on the intricate molecular mechanisms underlying maize development and stress responses.

This complex plays a pivotal role in regulating the production of anthocyanin pigments which contribute to the vibrant colors observed in many plants. However, certain gene products such as A3 and In1 act as inhibitors of this process. They achieve this by engaging in competitive interactions with the bHLH component of the MBW complex (Yan et al., 2021). These interactions lead to the suppression of anthocyanin accumulation ultimately influencing the coloration and pigmentation patterns in plants. This intricate regulatory mechanism highlights the importance of a fully functional MBW complex in orchestrating anthocyanin synthesis and underscores the significance of its precise regulation of plant physiology and coloration. Anthocyanin accumulation in maize crops transforms their vegetative tissues aleurone and pericarp into a captivating purple hue while the anthers take on a vivid shade of red (Peniche-Pavía et al., 2022). This stunning display of color not only enhances the visual appearance of maize plants but also showcases the fascinating role of anthocyanins in both the ornamental and biological aspects of these crops. Different environmental elements, including ultraviolet (UV) light and cold temperatures as well as phytohormones, including jasmonic acid (JA), salicylic acid (SA) and abscisic acid (ABA) have been found to be important determinants of this process.These elements act in concert to augment expression of the MBW complex thereby promoting anthocyanin production. In contrast, gibberellins (GAs) exert an opposing effect by downregulating the transcription of this tripartite complex (Wani et al., 2016). This intricate regulatory network underscores how plants adapt and respond to their surroundings by fine-tuning the synthesis of anthocyanins in response to changing environmental conditions and hormonal signals. To activate the genes involved in the biosynthesis of anthocyanins, complete MBW is required. A3 and In1 are two examples of gene products that compete with the bHLH members of this transcriptional complex to decrease the accumulation of anthocyanins.

This intricate regulatory mechanism highlights the importance of a fully functional MBW complex in orchestrating anthocyanin synthesis and underscores the significance of its precise regulation of plant physiology and coloration (Yan et al., 2021). Anthocyanin accumulation in maize crops transforms their vegetative tissues, aleurone and pericarp into a captivating purple hue while the anthers take on a vivid shade of red (Peniche-Pavía et al., 2022). This stunning display of color not only enhances the visual appearance of maize plants, but also showcases the fascinating role of anthocyanins in both the ornamental and biological aspects of these crops.

## 3 Anthocyanin catabolism

Anthocyanin catabolism in plants is a complex biochemical pathway responsible for the degradation of anthocyanin pigments which impart red, purple, and blue colors to various plant tissues, including fruits, flowers and leaves (Zhao et al., 2021). This degradation process involves a series of enzymatic reactions influenced by environmental factors and plant developmental stages. Initially anthocyanins undergo decarboxylation catalyzed by anthocyanin synthases resulting in the formation of colorless or pale-yellow flavanones. Subsequently, these flavanones can be transformed into chalcones through enzymatic action (Enaru et al., 2021). These compounds continue to break down into smaller colorless molecules through various reactions including isomerization and additional enzymatic modifications. These breakdown products may be transported within the plant or stored in cellular compartments (Hörtensteiner and Kräutler, 2011). The specific enzymes and genes involved can vary among plant species depending on environmental conditions such as light temperature, and nutrient availability which play a role in regulating this pathway (Li et al., 2020a; Jan et al., 2021). Anthocyanin catabolism is essential for resource recycling and allocation, particularly during periods of stress or when plants need to prioritize other metabolic processes (Dabravolski and Isayenkov, 2023).

Anthocyanin catabolism involves a complex interplay of molecular players. BHLH transcription factors regulate gene expression, influencing anthocyanin degradation while MYB transcription factors negatively impact synthesis genes leading to degradation. Enzymes like PPOs peroxidases, and laccases contribute by oxidizing and catalyzing breakdown with glucosidases destabilizing anthocyanins by removing sugar moieties. PAL influences precursor availability and CYP75 enzymes hydroxylate anthocyanins affecting their stability. Altogether, this orchestrated process highlights the intricate regulatory mechanisms governing anthocyanin catabolism (**Table 2**).

**Table 2:** Genes/Enzyme involved in Anthocyanin catabolism.

Gene/Enzyme Group	Function in Anthocyanin Breakdown	Reference
BHLH Transcription Factors	Regulate genes for anthocyanin degradation, modulating gene expression	(Qian et al., 2021)
MYB Transcription Factors	Negatively regulate anthocyanin synthesis genes, leading to degradation	(Li et al., 2022)
Polyphenol Oxidases (PPOs)	Oxidize anthocyanins, contributing to pigment breakdown	(Marszałek et al., 2017)
Peroxidases	Catalyze the breakdown of anthocyanins, often in stress conditions	(Zhao et al., 2021)
Laccases	Oxidize a range of phenolic compounds including anthocyanins	(Giménez et al., 2023)
Glucosidases	Remove sugar moieties from anthocyanins, destabilizing them	(Enaru et al., 2021)
Phenylalanine Ammonia-Lyase (PAL)	Influences precursor availability for anthocyanin synthesis	(Liu A. et al., 2023)
R2R3-MYB Transcription Factors	Downregulate biosynthesis genes, reducing anthocyanin levels	(Li et al., 2022)
CYP75 Family Enzymes	Involved in the hydroxylation of anthocyanins, affecting their stability	(Castellarin et al., 2006)
Flavonoid 3-O-Glucosyltransferase	Modify anthocyanin molecules, affecting their solubility and stability	(Sun et al., 2016)
Anthocyanidin Reductase (ANR)	Converts anthocyanidins to their corresponding aldehydes	(Liu et al., 2018)
Flavonol Synthase (FLS)	Competes for common substrates with anthocyanin biosynthetic enzymes	(Kumari et al., 2023)
Naringenin-Chalcone Synthase	Catalyzes early steps in flavonoid biosynthesis, influencing anthocyanin levels	(Gu et al., 2019)

## 4 Classification of anthocyanin

Important subgroup of flavonoids that is categorized as a polyphenol is called anthocyanins which is a phrase derived from the Greek words “Anthos” (flower) and “Kyanous” (dark blue). These soluble pigments play a crucial role in imparting distinctive hues to plants spanning from pink and red to purple shades. This is clearly observed in a range of fruits and vegetables such as berries, red apples, cherries, red grapes, red lettuce, eggplants, onions, and red cabbage all of which display these vibrant colors (Cappellini et al., 2021).

Anthocyanins are primarily divided into two categories based on their molecular structure flavonoids and phenolics. Flavonoids, a type of secondary metabolite are responsible for creating a broad spectrum of colors in various plant parts such as seeds, leaves, fruits, and flowers. On the other hand, phenolics are more commonly found in other plant tissues. The presence of different substitution patterns in various plant species results in a diverse array of anthocyanins. This includes types like 5-methoxycyanthocyanidins, 3-deoxyanthocyanidins, 7-methoxycyanthocyanidins, and 6-hydroxyanthocyanidins (**Figure 3**). This diversity demonstrates the complexity and pervasiveness of anthocyanins in the terrestrial plant ecosystem in addition to adding to the range of colors found in the plant kingdom (Mattioli et al., 2020).

**Figure 4:**
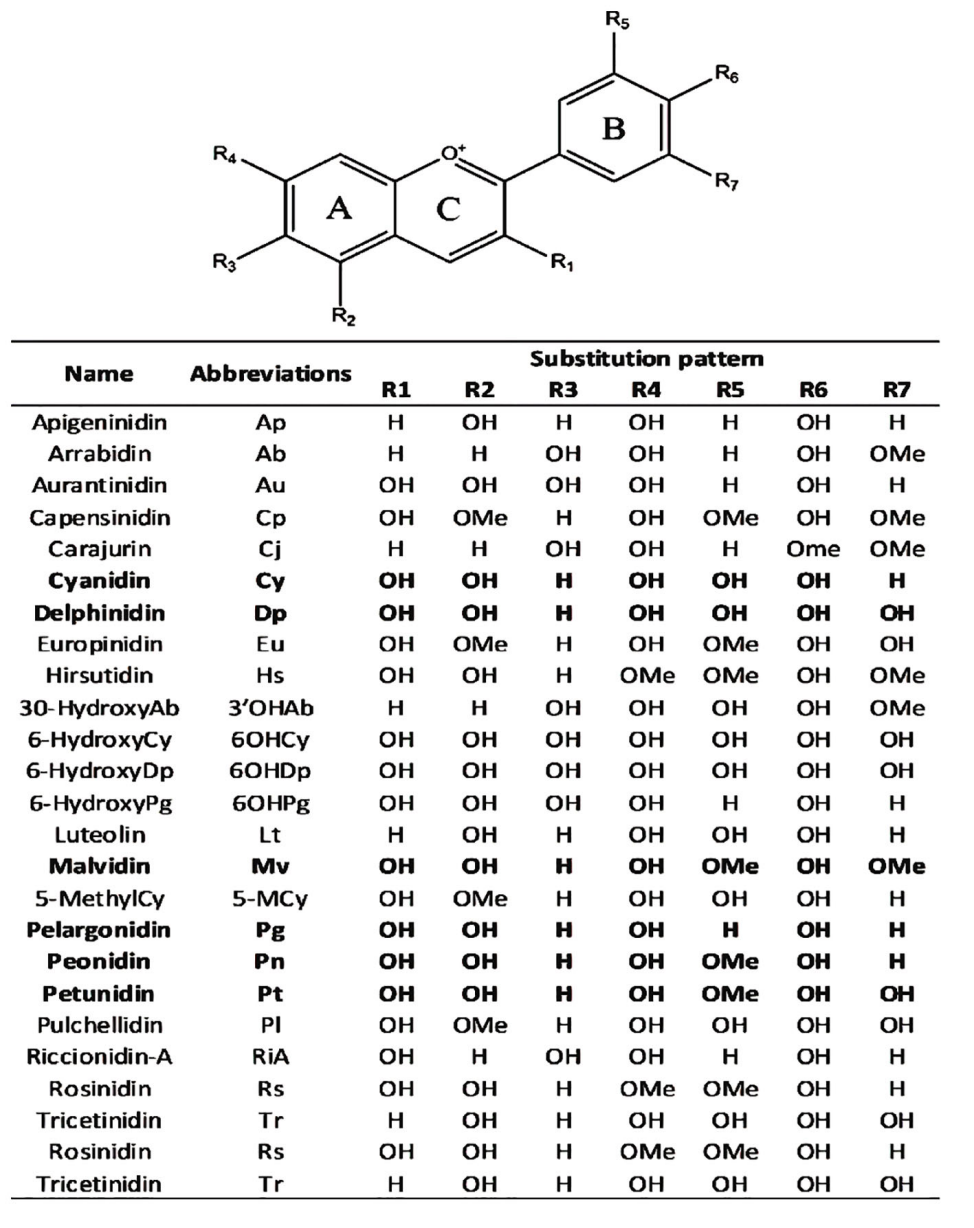
Chemical scaffolds of anthocyanins and their relative substituents. The most common anthocyanidins are reported in bold (Mannino et al., 2021).

Anthocyanidins are colored molecules of medium size that belong to the class of flavonoids (Brouillard, 1982). The geographical region known as the Andes in South America is recognized as the original source of purple corn. Extensive analysis has been conducted on the anthocyanins contained in various components of Andean purple corn, including its flowers, leaves, cobs, and kernels. The primary anthocyanins identified in these samples were cyanidin-3-dimalonylglucoside, cyanidin-3-glucoside, pelargonidin-3-glucoside, peonidin-3-glucoside, and related malonated derivatives (Cai et al., 2023). Overall, 25 anthocyanidins were identified (**Figure 4**). These anthocyanidins exhibit variations in their chemical structures due to the presence of hydroxyl (−OH) and methoxy (−OCH3) groups attached to the central scaffold core, as indicated in **Figure 4** (Mannino et al., 2021). Anthocyanidins are classified into three categories:3-hydroxyanthocyanidins, 3-deoxyanthocyanidins, and O-methylated anthocyanidins. Cyanidin (Cy), Cyanidin and its glycosides as part of the broader group of anthocyanins play significant functions in the physiology and propagation of plants. They observe pollinators and seed dispersers and function as defensive mechanisms against biotic and abiotic stressors. An important quality feature that draws in customers is the vibrant character of anthocyanins such as cyanidin. Because of these chemicals’ anti-inflammatory, antioxidant, neuroprotective and anti-diabetic qualities they are linked to a number of health advantages. Chemically speaking anthocyanins which include cyanidin are derivatives of the flavylium cation that are polyhydroxy and polymethoxy. They can have acylated moieties or sugar groups attached at various points (Mattioli et al., 2020). Delphinidin (Dp), Delphinidin in plants imparts vibrant colors attracting pollinators and aiding in reproduction. It protects against UV damage and oxidative stress with its antioxidant properties. Additionally, it plays a role in defense mechanisms and temperature adaptation and Pelargonidin (Pg), Pelargonidin in plants imparts red and orange colors aiding in pollination and seed dispersal. It offers protection against environmental stress with its antioxidant properties. Additionally it plays a role in the plant’s response to various stresses these are three of the most prevalent non-methylated anthocyanidins found in the natural environment. According to (Mannino et al., 2021). it was approximated that 50% of plant species that synthesize anthocyanidins possess the Cy gene, 12% possess the Dp gene, and 10% possess the Pg gene. Peonidin (Pn), malvidin (Mv) and petunidin (Pt), classified as methylated anthocyanidins are commonly present in several plant species (Saigo et al., 2020; Daniel et al., 2023).

Anthocyanidins are usually conjugated with sugar moieties leading to the synthesis of the corresponding anthocyanins. Following the addition of the sugar moiety at the 3rd and/or 5th positions (R1 and/or R2 substituents) of the chemical structure shown in **Figure 4** of the scaffold enzymatic glycosylation generally occurs. Because of glycosylation and anthocyanins have higher water solubility and stability than their related anthocyanidins. (Saigo et al., 2020). The primary stage of glycosylation usually sees the fusion of monosaccharides, specifically glucose, galactose, rhamnose, arabinose, rutinose and xylose. On certain occasions this stage may also feature the addition of disaccharides and trisaccharides (Rabanal-Atalaya and Medina-Hoyos, 2021; Qi et al., 2022).

Anthocyanins commonly initiate acylation with organic acids such as p-coumaric, caffeic, and ferulic acids through ester linkages, generally at the 3-position of the sugar moiety (colors, R. B.-A. as food, & 1982, 1982; Dini et al., 2018). Accordingly a considerable number of over 500 distinct anthocyanins have been identified thus far showing variations in both the glycosylation pattern of the underlying structure and the presence and location of aliphatic or aromatic carboxylates (Mannino et al., 2021). The anthocyanins produced by Cy, Dp, and Pg are the most widely distributed in plants and have a wide range of structural variations. Eighty percent of the leaves, 69 percent of the fruits, and fifty percent of the colored flowers include them (Kong et al., 2003; Rabanal-Atalaya and Medina-Hoyos, 2021). However, the distribution of anthocyanins produced by Pt, Mv, and Pn is restricted (Rabanal-Atalaya and Medina-Hoyos, 2021) The chemical scaffold’s conjugated bonds are one of the elements responsible for the light absorption at about 500 nm (Dini et al., 2018). However other factors that could affect color variation include the type of substituents found in the benzyl ring, the pH of the area, the state of aggregation, and complexation with other inorganic and organic molecules. Anthocyanins, for instance, have been found to exhibit a nearly full chromatic range (Dini et al., 2018).

## 5 Future prospects of anthocyanin & conclusions

In the realm of agricultural research the prospects of this study on anthocyanin biosynthesis in maize are both promising and far-reaching. As our understanding of the molecular mechanisms governing anthocyanin accumulation in fruit corn deepens, we stand at the ice of numerous exciting possibilities. First, the potential to enhance the nutritional and medicinal properties of fruit corn cannot be overstated. With anthocyanins known for their antioxidant and anti-inflammatory properties the development of genetically improved maize varieties with elevated anthocyanin content holds promises for creating healthier and nutrient-rich corn products for both human consumption and medicinal applications. Moreover, newfound knowledge about key regulatory genes, such as ZmCOP1 and ZmHY5, opens doors to precise genetic manipulation. This could enable us to fine-tune anthocyanin levels in maize, catering to specific preferences for color, flavor and nutritional benefits. The ability to produce corn varieties with enhanced color traits not only appeals to consumers but also has applications in the food industry, where natural colorants are highly sought after as healthier alternatives to synthetic additives.

Furthermore, this research paves the way for a deeper exploration of the intricate relationship between anthocyanin biosynthesis and other physiological processes in maize. Understanding how these compounds interact with sugar-to-starch conversion, as regulated by Sh2 can lead to the development of maize varieties with improved kernel quality and nutritional content. This approach has the potential to address nutritional deficiencies and food security concerns in regions where maize is a staple crop. In addition to its agronomic implications, this study’s insights into the molecular identification of genes involved in the anthocyanin pathway of maize lay the groundwork for broader applications in plant biology and biotechnology. These findings could inspire similar research in other crops, potentially unlocking novel pathways to produce valuable phytochemicals and contributing to more sustainable and resilient agriculture.

Future prospects emerging from this research, not only hold the key to improving the nutritional quality of maize, but also offer a window into the exciting possibilities of precision agriculture, sustainable food production, and the development of healthier and more vibrant crops. As we continue to delve deeper into the molecular mechanisms of anthocyanin biosynthesis in maize, the potential benefits to both human health and the agricultural industry are indeed promising and boundless.

In summary our review sheds light on the intricate molecular pathways involved in anthocyanin biosynthesis in maize (corn) identifying key genes, such as C1, C2, Pl1, Pl2, Sh2, ZmCOP1, and ZmHY5, which play pivotal roles in the accumulation of beneficial anthocyanin compounds, such as cyanidin-3-O-glucoside, perlagonidin-3-O-glucoside, and peonidin 3-O-glucoside. Notably, the Sh2 gene also influences sugar-to-starch conversion, affecting kernel quality and nutritional content, whereas ZmCOP1 and ZmHY5 are integral in controlling light responses and photomorphogenesis in maize. These findings not only advance our understanding of anthocyanin biosynthesis but also open avenues for the development of genetically improved maize varieties. By enhancing color traits and maximizing potential health benefits, such advancements could have broad implications for both the edible and medicinal applications of maize. Further research is imperative to deepen our understanding of the full potential of anthocyanins in maize for nutritional and health benefits.

## Author contributions

ZC: Conceptualization, Formal analysis, Investigation, Methodology, Writing – original draft, Writing – review & editing. RL: Formal analysis, Investigation, Supervision, Conceptualization, Data curation, Methodology, Writing – original draft, Writing – review & editing. NA: Software, Writing – review & editing. ML: Conceptualization, Writing – review & editing. SC: Conceptualization, Software, Formal analysis, Methodology, Writing – review & editing. NP: Conceptualization, Resources, Writing – review & editing. YQ: Funding acquisition, Methodology, Project administration, Resources, Supervision, Validation, Writing – original draft, Writing – review & editing.
